# 

**Published:** 1895-03

**Authors:** 


					CONTENTS.
Extra-peritoneal Closure of Artificial
Anus and Fsecal Fistula.
By J.
VjREIG bMITH, M.13., r.K.b.Jb.
Cystotomy for Bladder Tumours of
Doubtful Malignancy.
By J. Paul
Bush, M.R.C.S. Eng.
The Administration of Nitrous Oxide
as a Preliminary to Ether Anses-
thesia.
By Bertram M. H. Rogers,
B.A., M.D., B.Ch. Oxon.
10
Some Clinical Notes on Membranous
Enteritis.
M.R T
M.B. Lond., F.R.C.S. Eng.
14
The Bacterioscopic Diagnosis of Diph-
theria.
By F. Wallis Stoddart,
F-I.C., F.C.S.
18
Progress of the Medical Sciences:
Medicine.
By Theodore Fisher,
M.D.
24
Surgery.
By W. M. Barclay
Ophthalmology.
By F. R. Cross.
Reviews of Books
37
Notes on Preparations for the Sick
Meetings of Societies
68
The Library of the Bristol Medico-
Chirurgical Society
73
Obituary
76
Presentation to Dr. J. G. Swayne
78
Scraps
79

				

